# Activity of ceftolozane/tazobactam, imipenem/relebactam and ceftazidime/avibactam against clinical Gram-negative isolates—SMART United States 2019–21

**DOI:** 10.1093/jacamr/dlad152

**Published:** 2024-01-12

**Authors:** James A Karlowsky, Sibylle H Lob, Karri A Bauer, John Esterly, Fakhar Siddiqui, Katherine Young, Mary R Motyl, Daniel F Sahm

**Affiliations:** IHMA, Schaumburg, IL 60173, USA; Department of Medical Microbiology and Infectious Diseases, Max Rady College of Medicine, University of Manitoba, Winnipeg, MB R3E 0J9, Canada; IHMA, Schaumburg, IL 60173, USA; Merck & Co., Inc., Rahway, NJ 07065, USA; Merck & Co., Inc., Rahway, NJ 07065, USA; Merck & Co., Inc., Rahway, NJ 07065, USA; Merck & Co., Inc., Rahway, NJ 07065, USA; Merck & Co., Inc., Rahway, NJ 07065, USA; IHMA, Schaumburg, IL 60173, USA

## Abstract

**Background:**

Ongoing national and international surveillance efforts are critical components of antimicrobial stewardship, resistance monitoring, and drug development programs. In this report, we summarize the results of ceftolozane/tazobactam, imipenem/relebactam, ceftazidime/avibactam and comparator agent testing against 10 509 Enterobacterales and 2524 *Pseudomonas aeruginosa* collected by USA clinical laboratories in 2019–21 as part of the SMART global surveillance programme.

**Methods:**

MICs were determined by CLSI broth microdilution and interpreted using 2023 CLSI M100 breakpoints.

**Results:**

Most Enterobacterales were ceftazidime/avibactam susceptible (>99%), meropenem susceptible (99%) and ceftolozane/tazobactam susceptible (94%). Non-Morganellaceae Enterobacterales were also highly susceptible to imipenem/relebactam (99%). Ceftolozane/tazobactam inhibited 94% of *Escherichia coli* and 89% of *Klebsiella pneumoniae* with ceftriaxone non-susceptible/non-carbapenem-resistant phenotypes. Against *P. aeruginosa*, ceftolozane/tazobactam (97% susceptible) was more active than ceftazidime/avibactam (95%) and imipenem/relebactam (91%). MDR and difficult-to-treat resistance (DTR) phenotypes were identified in 13% and 7% of *P. aeruginosa* isolates, respectively. Ceftolozane/tazobactam remained active against 78% of MDR *P. aeruginosa* (13% and 23% higher than ceftazidime/avibactam and imipenem/relebactam, respectively) and against 74% of DTR *P. aeruginosa* (24% and 37% higher than ceftazidime/avibactam and imipenem/relebactam, respectively). Length of hospital stay at the time of specimen collection, ward type and infection type resulted in percent susceptible value differences of >5% across isolate demographic strata for some antimicrobial agent/pathogen combinations.

**Conclusions:**

We conclude that in the USA, in 2019–21, carbapenem (meropenem) resistance remained uncommon in Enterobacterales and ceftolozane/tazobactam was more active than both ceftazidime/avibactam and imipenem/relebactam against *P. aeruginosa*.

## Introduction

Ongoing national and international surveillance efforts that generate and publish current data are critical components of antimicrobial stewardship, resistance monitoring and drug development programmes. CLSI reference method testing and reporting of the *in vitro* activities of newer parenteral antimicrobial agents such as the β-lactam/β-lactamase inhibitor combinations ceftolozane/tazobactam, imipenem/relebactam and ceftazidime/avibactam is important because these agents may not be tested routinely in clinical laboratories given their use may be reserved for selected patients or they may not be included on the panels/cards used by laboratories for routine automated antimicrobial susceptibility testing. The absence of routine testing of newer agents risks prospective identification of emergent unanticipated anomalies in susceptibility.

Briefly, ceftolozane/tazobactam is a novel antipseudomonal cephalosporin partnered with an established β-lactamase inhibitor. Imipenem/relebactam combines a recognized carbapenem/renal dehydropeptidase inhibitor (cilastatin) and relebactam, a non-β-lactam diazabicyclooctane (DBO) inhibitor of most Ambler class A β-lactamases (including ESBLs and KPCs) and class C β-lactamases (AmpC). Ceftazidime/avibactam also combines a DBO inhibitor of class A, C and some D (OXA-48-like) β-lactamases with an established cephem.

Ceftolozane/tazobactam was approved by the US FDA in 2014, ceftazidime/avibactam in 2015 and imipenem/relebactam in 2019. In the USA, each of the three agents is indicated for the treatment of complicated intraabdominal infection, complicated urinary tract infection and hospital-acquired bacterial pneumonia and ventilator-associated bacterial pneumonia caused by susceptible Gram-negative microorganisms.^1–3^ IDSA currently lists imipenem/relebactam and ceftazidime/avibactam as preferred or alternative treatments (depending on infection source) for carbapenem-resistant Enterobacterales (CRE) infection.^4^ IDSA also recommends ceftolozane/tazobactam, imipenem/relebactam and ceftazidime/avibactam as preferred options for the treatment of serious infections (suspected or documented) caused by *Pseudomonas aeruginosa* with a difficult-to-treat resistance (DTR) phenotype.^4,5^ Currently, CRE, and consequently DTR Enterobacterales, remain relatively uncommon in the USA (<2%),^5–7^ in contrast to some other countries, while *P. aeruginosa* with DTR and MDR phenotypes are common in many countries, including the USA.^7–13^

The current report provides an update on antimicrobial susceptibility testing results for Enterobacterales and *P. aeruginosa* isolates submitted to the SMART (Study for Monitoring Antimicrobial Resistance Trends) global surveillance programme from 2019 to 2021 by participating medical centre laboratories in the USA.

## Materials and methods

### Bacterial isolates

In 2019–21 the SMART global surveillance programme requested that each participating medical laboratory collect consecutive isolates of Gram-negative bacilli from bloodstream infections (50 isolates/year), intra-abdominal infections (50 isolates/year), lower respiratory tract infections (100 isolates/year) and urinary tract infections (50 isolates/year). Only one isolate per patient per species per year was accepted. Isolates were collected without regard to specific species quotas. Twenty-four medical centre laboratories in 16 states in the USA collected 14 177 isolates of Gram-negative bacilli in 2019–21 as part of the SMART global surveillance programme (Table S1, available as Supplementary data at *JAC-AMR* Online). Sixteen of the 24 medical centre laboratories participated in all 3 years of isolate collection, four laboratories in 2 years, and four laboratories in 1 year. The isolates collected by the combined 24 medical centre laboratories in 2019–21 comprised 2937 bloodstream infection isolates (20.8% of all isolates), 2377 intra-abdominal infection isolates (16.8%), 5706 lower respiratory tract infection isolates (40.4%) and 3036 urinary tract infection isolates (21.5%); for 61 isolates (0.4% of all isolates) the infection source was not specified (Table S2). All isolates were sent to IHMA (Schaumburg, IL, USA) where organism identity was confirmed using MALDI-TOF MS (Bruker Daltonics, Billerica, MA, USA) and antimicrobial susceptibility testing was performed.

### Antimicrobial susceptibility testing

MICs were determined using the CLSI broth microdilution method on in-house prepared panels at IHMA and interpreted using 2023 CLSI breakpoints.^14,15^ Avibactam was obtained from Advanced ChemBlocks, Inc., (Burlingame, CA, USA), ceftolozane, imipenem and relebactam from Merck & Co., Inc., (Rahway, NJ, USA) and tazobactam from USP. Other antimicrobial agents were purchased from commercial sources.

A ceftriaxone non-susceptible/non-CRE phenotype was defined for *Escherichia coli* and *Klebsiella pneumoniae* isolates testing with a ceftriaxone MIC of ≥2 mg/L and an ertapenem MIC of ≤0.5 mg/L and was intended to approximate the subset of ESBL-producing, carbapenem-susceptible isolates amongst all isolates of those two species. MDR was defined as an isolate testing as resistant to ≥3 sentinel drugs: amikacin, aztreonam, cefepime, colistin, imipenem, levofloxacin and piperacillin/tazobactam. DTR was defined as an isolate testing as non-susceptible (intermediate or resistant) to both β-lactams (aztreonam, cefepime, ceftazidime, imipenem, meropenem, piperacillin/tazobactam) and fluoroquinolones (levofloxacin).^5^ The definition of DTR excluded antimicrobial susceptibility testing results for ceftolozane/tazobactam, imipenem/relebactam and ceftazidime/avibactam.

Morganellaceae (*Morganella*, *Proteus*, *Providencia*) were excluded from susceptibility analyses for imipenem/relebactam because the CLSI breakpoints for that agent do not apply to the family Morganellaceae.^14^ The CLSI M100 document also states that Morganellaceae are intrinsically less susceptible or resistant to imipenem (compared with other species of Enterobacterales) by a mechanism independent of β-lactamase production;^14,16^ relebactam would not be expected to enhance imipenem’s activity against isolates of Morganellaceae.

Statistical analyses of percent susceptible values across isolate-associated strata [e.g. hospital length of hospital stay (LOS), ward type, infection type] was not performed because of the large number of isolates tested where differences in percent susceptible values of <1% are statistically significant by χ*^2^* testing.

## Results

Enterobacterales (*n *= 10 509; 74.4% of all isolates), including non-Morganellaceae Enterobacterales (NME) (*n *= 9524; 67.5%) and *P. aeruginosa* (*n *= 2524; 17.9%) accounted for 92% of the 14 117 Gram-negative bacilli collected by the 24 US medical centre laboratories participating in the SMART global surveillance programme from 2019 to 2021 (Table S3).

Almost all Enterobacterales isolates were susceptible to meropenem (99.0%) and ceftazidime/avibactam (99.8%); 94.2% of isolates were ceftolozane/tazobactam susceptible; 85%–89% of isolates were susceptible to imipenem, piperacillin/tazobactam, cefepime, ceftazidime and aztreonam; and 80% of isolates were levofloxacin susceptible (Table 1). Most ceftazidime non-susceptible (98.9%) and meropenem non-susceptible (90.2%) isolates of Enterobacterales were ceftazidime/avibactam susceptible. Isolates of NME were highly susceptible to both imipenem/relebactam (98.9%) and imipenem (94.9%); and most ceftazidime non-susceptible (98.4%) isolates and many meropenem non-susceptible (76.0%) of NME were imipenem/relebactam susceptible.

**Table 1.** Antimicrobial susceptibility testing results for isolates of Enterobacterales, NME and *P. aeruginosa*, including resistant isolate subsets, collected by the SMART global surveillance programme in the USA from 2019 to 2021

Organism/phenotype^a^	*n*	Susceptible (%)										
		C/T	IMI/REL	CZA	IMI	MEM	P/T	FEP	CAZ	ATM	LVX^b^	AMK^c^
Enterobacterales	10 509	94.2	NA	99.8	88.4	99.0	87.8	89.0	85.2	85.0	79.9	NA
CAZ-NS	1552	62.5	NA	98.9	86.7	93.8	43.6	36.1	0	10.2	45.7	NA
MEM-NS	102	6.9	NA	90.2	4.9	0	2.9	7.8	5.9	5.9	25.5	NA
NME	9524	93.8	98.9	99.9	94.9	99.0	86.9	88.3	84.5	83.7	81.2	NA
CAZ-NS	1478	61.7	98.4	99.1	89.9	93.6	41.9	34.6	0	7.1	46.4	NA
MEM-NS	100	6.0	76.0	90.0	5.0	0	2.0	7.0	5.0	5.0	26.0	NA
*E. coli*	4154	98.3	99.9	100	99.5	99.8	92.7	86.2	86.3	85.1	71.5	NA
CRO NS/non-CRE	706	94.2	100	100	100	100	79.3	24.5	26.9	19.5	25.1	NA
CAZ-NS	571	88.1	99.6	99.8	97.7	98.4	71.8	16.6	0	6.7	22.6	NA
*K. pneumoniae*	1930	95.6	99.1	99.7	96.8	97.9	85.6	85.6	84.9	84.7	82.8	NA
CRO NS/non-CRE	274	88.7	99.6	100	98.9	100	60.9	19.3	16.8	13.1	39.1	NA
CAZ-NS	291	71.8	96.2	98.3	84.5	85.9	44.0	12.4	0	6.2	31.3	NA
*P. aeruginosa*	2524	96.6	90.7	94.8	65.1	79.4	78.6	82.3	80.7	71.1	67.0	97.0
CAZ-NS	487	83.0	73.1	73.1	37.6	47.2	12.9	21.8	0	14.6	42.8	91.6
MEM-NS	520	88.7	56.2	79.6	5.6	0	41.3	48.7	50.6	27.5	27.6	91.7
MDR	337	78.3	55.5	65.6	15.7	23.4	8.9	13.1	14.2	4.5	21.1	85.8
DTR	167	73.7	36.5	49.7	0	0	0	0	0	0	0	83.8
Enterobacterales + *P. aeruginosa*	13 033	94.7	NA	98.9	83.8	95.2	86.0	87.7	84.4	82.3	77.4	NA
NME +* P. aeruginosa*	12 048	94.4	97.2	98.8	88.7	94.9	85.1	87.1	83.7	81.1	78.2	NA

C/T, ceftolozane/tazobactam; IMI/REL, imipenem/relebactam; CZA, ceftazidime/avibactam; IMI, imipenem; MEM, meropenem; P/T, piperacillin/tazobactam; FEP, cefepime; CAZ, ceftazidime; ATM, aztreonam; LVX, levofloxacin; AMK, amikacin; NME, non-*Morganellaceae* Enterobacterales; CRO, ceftriaxone; CRE, carbapenem-resistant Enterobacterales; DTR, difficult-to-treat resistance; NA, not available; NS, non-susceptible.

^a^Too few isolates of *E. coli* (*n *= 10) and *K. pneumoniae* (*n *= 41) with a meropenem-non-susceptible phenotype were identified to be included in the table.

^b^Susceptibility to levofloxacin was not available for *Salmonella* spp. (*n *= 17) because the tested concentration range did not extend low enough for the *Salmonella*-specific CLSI susceptible breakpoint for that agent.

^c^Susceptibility to amikacin was not determinable for Enterobacterales because the tested concentration range did not extend low enough to include the revised 2023 CLSI susceptible breakpoint for that agent.

Seventeen percent of *E. coli* and 14% of *K. pneumoniae* isolates had a ceftriaxone non-susceptible/non-CRE phenotype; 99%–100% of ceftriaxone non-susceptible/non-CRE *E. coli* and ceftriaxone non-susceptible/non-CRE *K. pneumoniae* were susceptible to imipenem/relebactam, imipenem, meropenem and ceftazidime/avibactam. Ceftolozane/tazobactam was more active than piperacillin/tazobactam against both ceftriaxone non-susceptible/non-CRE *E. coli* (94.2% susceptible versus 79.3%) and ceftriaxone non-susceptible/non-CRE *K. pneumoniae* (88.7% susceptible versus 60.9%). Only 25.1% of ceftriaxone non-susceptible/non-CRE *E. coli* and 39.1% of ceftriaxone non-susceptible/non-CRE *K. pneumoniae* were levofloxacin susceptible. Ninety-eight percent or more of ceftazidime non-susceptible *E. coli* were susceptible to imipenem, meropenem, imipenem/relebactam and ceftazidime/avibactam; and most ceftazidime non-susceptible *K. pneumoniae* were susceptible to imipenem/relebactam (96.2%) and ceftazidime/avibactam (98.3%) but were less susceptible to imipenem and meropenem (85%–86%). MDR and DTR analyses were not performed for Enterobacterales or NME given that 99% of isolates were meropenem susceptible. Imipenem/relebactam (96.1% susceptible) and ceftazidime/avibactam (97.6%) inhibited most ceftolozane/tazobactam non-susceptible isolates of NME (*n *= 587), while ceftazidime/avibactam inhibited 92.5% of imipenem/relebactam non-susceptible isolates (*n* = 106) and imipenem/relebactam inhibited 42.9% of ceftazidime/avibactam-resistant isolates (*n *= 14) (Table S4).

Meropenem percent non-susceptible values (i.e., CRE) were ≤2% for both Enterobacterales and NME across hospital LOS, ward type and infection type strata (Table 2). Percent susceptible values for ceftolozane/tazobactam, piperacillin/tazobactam, cefepime, ceftazidime and aztreonam were >5% higher for Enterobacterales isolates from patients with LOS <48 h than ≥48 h. Percent susceptible values for piperacillin/tazobactam were also ≥5% higher for isolates from patients hospitalized in non-ICU wards than in ICU wards. Percent susceptible values for ceftolozane/tazobactam, imipenem, piperacillin/tazobactam, aztreonam and levofloxacin varied by ≥5% across the four infection type isolates tested. Percent susceptible values were lower for lower respiratory tract isolates than for isolates from other infection types for all agents tested except levofloxacin. Against NME, imipenem/relebactam percent susceptible values varied by <2% for isolates stratified by hospital LOS, ward type and infection type. Across the four US census regions, only percent susceptible values for aztreonam against Enterobacterales differed by >5% (82.0% in the West compared with 87.1% in the Northeast and 87.2% in the Midwest (Table S5).

**Table 2.** Antimicrobial susceptibility testing results for isolates of Enterobacterales, NME, and *P. aeruginosa* collected by the SMART global surveillance program in the United States from 2019 to 2021 stratified by length of hospital stay at the time of specimen collection, ward type, and infection type

		% Susceptible										
Organism/stratum^a^	*n*	C/T	IMR	CZA	IPM	MEM	TZP	FEP	CAZ	ATM	LVX^b^	AMK^c^
Enterobacterales												
LOS (h)												
≥48	3030	90.5	NA	99.6	87.3	98.3	81.6	85.0	79.5	79.0	79.5	NA
<48	5895	96.0	NA	99.9	89.0	99.4	90.7	90.6	87.7	87.4	80.0	NA
Ward type												
ICU	2526	91.3	NA	99.8	88.0	98.3	83.6	87.5	82.2	81.6	80.8	NA
Non-ICU	5691	94.8	NA	99.8	88.0	99.3	88.5	89.1	85.6	85.6	79.2	NA
Infection type												
BSI	2598	96.3	NA	100	90.9	99.3	91.0	89.2	86.9	86.8	76.5	NA
IAI	2088	94.3	NA	99.8	91.4	99.3	87.5	90.8	86.5	86.3	81.7	NA
LRTI	3082	91.0	NA	99.7	85.1	98.1	82.9	86.8	82.0	81.8	81.9	NA
UTI	2692	95.8	NA	99.9	87.4	99.6	90.6	89.9	86.2	85.6	79.4	NA
NME												
LOS (h)												
≥48	2827	90.1	98.2	99.7	92.3	98.2	80.7	84.5	78.8	77.9	80.2	NA
<48	5283	95.7	99.2	99.9	96.3	99.4	89.9	89.9	86.9	86.2	81.5	NA
Ward type												
ICU	2338	90.9	98.5	99.8	92.9	98.2	82.7	87.1	81.7	80.6	82.6	NA
Non-ICU	5115	94.4	99.0	99.9	95.2	99.2	87.5	88.3	84.8	84.1	80.6	NA
Infection type												
BSI	2368	96.1	99.4	100	96.9	99.4	90.3	88.4	86.4	85.8	77.5	NA
IAI	1951	94.0	99.4	99.8	96.3	99.2	86.8	90.2	86.1	85.4	81.8	NA
LRTI	2826	90.4	97.8	99.7	90.8	97.9	81.9	86.6	81.5	80.8	84.1	NA
UTI	2336	95.5	99.3	99.9	96.8	99.6	89.5	88.8	84.8	83.7	80.9	NA
*P. aeruginosa*												
LOS (h)												
≥48	922	95.9	88.9	93.6	62.1	77.2	75.1	80.4	77.4	67.7	69.2	98.0
<48	1248	96.7	91.8	95.4	66.7	81.2	81.3	83.3	83.7	73.6	64.9	96.2
Ward type												
ICU	791	96.3	90.0	94.6	62.7	77.6	75.7	81.4	78.8	69.4	69.2	98.0
Non-ICU	1325	96.8	90.9	94.8	66.6	81.0	80.2	82.1	81.2	72.2	66.0	96.5
Infection type												
BSI	223	99.6	95.1	97.8	77.1	87.0	86.5	91.9	89.2	79.8	76.2	98.2
IAI	233	97.0	88.4	95.3	63.9	80.7	85.4	88.4	86.7	80.7	76.0	97.9
LRTI	1763	95.8	89.8	94.1	62.6	76.9	75.7	79.5	77.8	68.1	64.3	96.6
UTI	294	99.0	94.6	96.6	73.5	87.8	84.7	88.1	86.7	75.5	70.4	98.6

C/T, ceftolozane/tazobactam; IMR, imipenem/relebactam; CZA, ceftazidime/avibactam; IPM, imipenem; MEM, meropenem; TZP, piperacillin/tazobactam; FEP, cefepime; CAZ, ceftazidime; ATM, aztreonam; LVX, levofloxacin; AMK, amikacin; LOS, LOS at time of specimen collection; BSI, bloodstream infection; IAI, intra-abdominal infection; LRTI, lower respiratory tract infection; UTI, urinary tract infection; NA, not available.

^a^Numbers of isolates in individual strata may not total to the numbers of isolates reported in Table 1 because some isolates do not have a specified LOS, patient location or infection type and some isolates were from emergency departments.

^b^Susceptibility to levofloxacin was not available for *Salmonella* spp. (*n *= 17) because the tested concentration range did not extend low enough for the *Salmonella*-specific CLSI susceptible breakpoint for that agent.

^c^Susceptibility to amikacin was not determinable for Enterobacterales because the tested concentration range did not extend low enough to include the revised 2023 CLSI susceptible breakpoint for that agent.

Ceftolozane/tazobactam and amikacin (both 97% susceptible) were the most active agents tested against *P. aeruginosa*, followed by ceftazidime/avibactam (95%) and imipenem/relebactam (91%); 79%–82% of isolates were susceptible to meropenem, piperacillin/tazobactam, cefepime and ceftazidime, while all other β-lactams and levofloxacin had percent susceptible values of 71% or less (Table 1). Amikacin was the most active agent (92% susceptible) against ceftazidime non-susceptible and meropenem non-susceptible isolate subsets of *P. aeruginosa*, followed by ceftolozane/tazobactam (83.0% and 88.7% susceptible, respectively).

**Figure 1.**
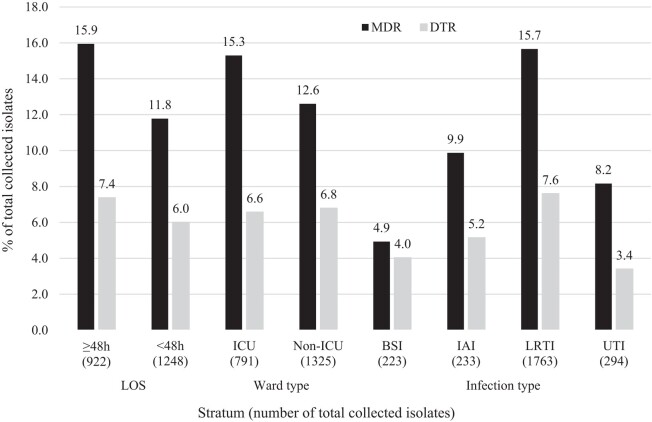
Prevalence of MDR and DTR phenotypes among isolates of *P. aeruginosa* collected by the SMART global surveillance programme in the USA from 2019 to 2021. LOS, LOS at time of specimen collection; BSI, bloodstream infection; IAI, intra-abdominal infection; LRTI, lower respiratory tract infection; UTI, urinary tract infection.

MDR and DTR phenotypes were identified in 13.4% and 6.6% of *P. aeruginosa* isolates, respectively (Table 1). The prevalence of MDR and DTR isolates of *P. aeruginosa* was higher among isolates collected from patients with hospital LOS ≥48 h than <48 h and MDR isolates were more prevalent among patients in ICU wards than non-ICU wards, while the DTR rate was similar (0.2% difference) in ICU and non-ICU wards (Figure 1). MDR and DTR phenotypes were most common among lower respiratory tract infection isolates (15.7% and 7.6%, respectively) and least common among bloodstream infection isolates (4.9% and 4.0%).

Ceftolozane/tazobactam remained active against 78.3% of MDR *P. aeruginosa* isolates: 7.5% lower than amikacin but 12.7% and 22.8% higher than ceftazidime/avibactam and imipenem/relebactam, respectively; and 55%–74% higher than other comparator β-lactams. Against DTR isolates, which by definition are not susceptible to any commonly used β-lactams (including carbapenems) and fluoroquinolones, susceptibility to ceftolozane/tazobactam was 10% lower than amikacin and 24% and 37% higher than ceftazidime/avibactam and imipenem/relebactam, respectively.

Among ceftolozane/tazobactam non-susceptible isolates of *P. aeruginosa*, the percent susceptible rate for imipenem/relebactam (51.8%) was 9% higher than for ceftazidime/avibactam (42.4%) (Table S6). The percent susceptible rate for ceftolozane/tazobactam was 14% higher than ceftazidime/avibactam against imipenem/relebactam non-susceptible isolates and 18% higher than imipenem/relebactam against ceftazidime/avibactam-resistant isolates. Ceftolozane/tazobactam retained activity against 62.6% of ceftazidime/avibactam-resistant *P. aeruginosa*, whereas only 42.4% of ceftolozane/tazobactam non-susceptible isolates were ceftazidime/avibactam susceptible.

Among *P. aeruginosa*, percent susceptible values for piperacillin/tazobactam, ceftazidime and aztreonam were >5% higher for isolates from patients with hospital LOS <48 h than ≥48 h (Table 2). Percent susceptible values for all agents differed by <5% between isolates from patients hospitalized in non-ICU wards and in ICU wards. Percent susceptible values for all agents except ceftolozane/tazobactam, ceftazidime/avibactam and amikacin varied by >5% across the four infection type isolates tested. Percent susceptible values were lower for lower respiratory tract isolates than isolates from other infection types for all agents tested except imipenem/relebactam (lowest for intra-abdominal infection isolates). Across the four US census regions, percent susceptible values for imipenem/relebactam, imipenem, meropenem, piperacillin/tazobactam, cefepime, ceftazidime, aztreonam and levofloxacin were >5% lower in the Northeast region than at least one other region (Table S5).

Against both Enterobacterales (or NME for imipenem/relebactam) and *P. aeruginosa* combined, 94%–95% of isolates were ceftolozane/tazobactam susceptible, 95% of isolates were meropenem susceptible, 97% of isolates were imipenem/relebactam susceptible and 99% of isolates were ceftazidime/avibactam susceptible (Table 1).

## Discussion

In the USA in 2019–21, meropenem resistance remained uncommon (1%) among clinical isolates of Enterobacterales (Table 1), including across LOS (i.e. community-acquired and hospital-acquired infection), ward type (ICU and non-ICU wards) and infection type (bloodstream, intra-abdominal, lower respiratory tract and urinary tract) strata (Table 2), confirming data published by other investigators surveying Enterobacterales isolates from medical centre laboratories across the USA in 2018–20^17,18^ and earlier.^19^ The majority of clinical isolates of meropenem non-susceptible Enterobacterales in the USA carry a KPC enzyme.^19^ The high percent susceptible values we observed for ceftazidime/avibactam (99.8%) and ceftolozane/tazobactam (94.2%) against Enterobacterales, and imipenem/relebactam against NME (98.9%) confirm earlier reports describing the consistent *in vitro* activities of these agents over time, including the inhibition of KPC by imipenem/relebactam and ceftazidime/avibactam.^7,17,18^ The higher percent susceptible value for ceftolozane/tazobactam (94.2%) compared with piperacillin/tazobactam (87.8%) against Enterobacterales isolates may reflect the presence of the OXA-1 penicillinase in some ESBL non-CRE isolates.^20^

In contrast to Enterobacterales, recent (2019–21) meropenem (79%) and imipenem (65%) percent susceptible values for US clinical isolates of *P. aeruginosa* (Table 1) preclude carbapenem use as empirical therapy when this pathogen is highly suspected. Cephalosporins, piperacillin/tazobactam, aztreonam and levofloxacin also tested with percent susceptible values ≤82% (Table 1). The current study found ceftolozane/tazobactam (97% susceptible) to be more active than both ceftazidime/avibactam (95%) and imipenem/relebactam (91%) against clinical isolates of *P. aeruginosa*, confirming an earlier report testing ICU and non-ICU isolates collected in the USA,^17^ and indicate that ceftolozane/tazobactam, imipenem/relebactam and ceftazidime/avibactam have largely maintained their potent *in vitro* activities against isolates from US patients since their release.^8–10,21–25^

Previous studies have also reported that imipenem/relebactam demonstrated greater activity than ceftazidime/avibactam against ceftolozane/tazobactam non-susceptible clinical isolates of *P. aeruginosa* collected in the USA,^13,18^ suggesting that both ceftolozane/tazobactam and imipenem/relebactam be considered for routine antimicrobial susceptibility testing of clinical isolates of *P. aeruginosa*. Our study (Table S6) confirmed this observation with more recent data. We also observed that ceftazidime/avibactam was more active than imipenem/relebactam against meropenem non-susceptible isolates of both *P. aeruginosa* and NME (Enterobacterales), which is not unexpected given the differences in cellular uptake of the two β-lactam constituents and differences in mechanisms of resistance for these two agents (Table 1).^7,8,13,26^

In the USA almost all (99%) of carbapenem, imipenem/relebactam or ceftolozane/tazobactam non-susceptible isolates of *P. aeruginosa* do not carry an acquired β-lactamase, including carbapenemases,^13,27^ in contrast to many regions in the world where carbapenemase rates among resistant *P. aeruginosa* isolates are higher.^28,29^ Lower ceftolozane/tazobactam, imipenem/relebactam and ceftazidime/avibactam susceptibilities have been reported in regions outside the USA in association with higher numbers of isolates carrying MBLs.^30^

Isolates of *P. aeruginosa* in the current study were also highly susceptible to amikacin (97.0%); however, aminoglycoside use is associated with well-established toxicities and therapeutic limitations, and the use of this agent class is strongly discouraged. Both the CLSI^14^ and EUCAST^31,32^ have recently introduced susceptibility testing and reporting restrictions for aminoglycosides to further dissuade their use.

Clinical isolates of *P. aeruginosa* with MDR phenotypes are common in the USA, particularly among isolates from patients hospitalized for ≥48 h, in ICUs and among lower respiratory tract infection isolates (Figure 1).^8–11,13^ The MDR rate in the current study was 13%, comparable to previously reported MDR rates.^8–11,13^ In the current study, 78% of MDR *P. aeruginosa* were ceftolozane/tazobactam susceptible compared with 66% ceftazidime/avibactam susceptible and 56% imipenem/relebactam susceptible. In the current study, 7% of *P. aeruginosa* isolates had a DTR phenotype; percent susceptible rates for DTR isolates exceeded 50% for only amikacin (84% susceptible) and ceftolozane/tazobactam (74% susceptible). Gram-negative bacilli with a DTR phenotype are associated with therapeutic failure and mortality, particularly in severely ill patients.^5,6^ The significant numbers of both MDR and DTR *P. aeruginosa*, as well as emerging resistance and MDR in all human pathogens, underscores the necessity for strong and consistent antimicrobial stewardship programmes across all patient types, with practices that are independent of hospital LOS, ward type or infection type.

The strengths of the current study are that it collected isolates from a relatively consistent set of medical centre laboratories in the USA for a recent 3 year period according to a consistent protocol that did not include species quotas and used reference broth microdilution antimicrobial susceptibility testing performed in a central laboratory to test three newer β-lactam/β-lactamase inhibitor combinations and eight established agents. Study limitations include that there was some change in study participation by individual medical centre laboratories over the 3 years of the study and that it used quotas to collect isolates from different infection types that may affect the overall estimates of resistance and β-lactamase prevalence. The data presented in this study may be limited by the small annual sample size (250 Gram-negative isolates per medical centre per year). The data generated from isolates submitted by participating medical centres within the USA should not be extrapolated to represent all isolates or geographical areas within the country.

We conclude that most clinical isolates of Enterobacterales (99%) in the USA remain meropenem susceptible, meaning most MDR isolates remain meropenem susceptible and DTR isolates are extremely rare (<1%). Ceftriaxone non-susceptible/non-CRE phenotype *E. coli* (17% of isolates) and *K. pneumoniae* (14%) were common but highly susceptible (≥99%) to imipenem, meropenem, imipenem/relebactam and ceftazidime/avibactam. Ceftolozane/tazobactam (97% susceptible) was the most active anti-pseudomonal β-lactam/β-lactamase inhibitor combination tested. Ceftolozane/tazobactam, imipenem/relebactam and ceftazidime/avibactam remain highly active against common Gram-negative pathogens and provide important treatment options for patients with infections caused by antimicrobial non-susceptible and -resistant isolates. Continued surveillance is warranted.

## Supplementary Material

dlad152_Supplementary_Data

## Transparency declarations

S.H.L. and D.F..S work for IHMA, which receives funding from Merck Sharp & Dohme LLC, a subsidiary of Merck & Co., Inc., Rahway, NJ, USA for the SMART surveillance programme. J.A.K. is a consultant to IHMA, and an employee of Shared Health Manitoba and the University of Manitoba. K.A.B., J.E., F.S., K.Y. and M.R.M. are employees of Merck Sharp & Dohme LLC, and own stock and options in Merck & Co., Inc., Rahway, NJ, USA. The IHMA authors do not have personal financial interests in the sponsor of this manuscript (Merck Sharp & Dohme LLC).

## Supplementary data

Tables S1 to S6 are available as Supplementary data at *JAC-AMR* Online.
