# Supplementary material for: Activity of ceftolozane/tazobactam, imipenem/relebactam and ceftazidime/avibactam against clinical Gram-negative isolates—SMART United States 2019–21
Source: JAC Antimicrob Resist. 2024 Jan 12;6(1):dlad152. doi: 10.1093/jacamr/dlad152 (PMC10786191; doi:10.1093/jacamr/dlad152)
Supplement: dlad152_Supplementary_Data [file dlad152_supplementary_data.docx]

**Supplemental Data**

**Table S1.** Summary of isolates of Gram-negative bacilli collected by 24 United States medical center laboratories participating in the SMART global surveillance program from 2019 to 2021 stratifed by geographic location and year of collection

|  |  |  | Year | | | |
| --- | --- | --- | --- | --- | --- | --- |
|  |  |  | Number of isolates | | | |
| Laboratory | State | United States census region | 2019 | 2020 | 2021 | Total |
| A | Illinois | Midwest | 247 | 249 | 250 | 746 |
| B | Illinois | Midwest | 248 | 250 | 248 | 746 |
| C | Indiana | Midwest | 247 | 250 |  | 497 |
| D | Indiana | Midwest | 251 | 198 | 246 | 695 |
| E | Michigan | Midwest | 250 | 250 | 250 | 750 |
| F | Nebraska | Midwest | 239 |  |  | 239 |
| G | Ohio | Midwest | 253 | 247 | 249 | 749 |
| H | Wisconsin | Midwest | 53 | 27 |  | 80 |
| I | Wisconsin | Midwest | 240 | 250 | 243 | 733 |
| J | New York | Northeast | 223 | 253 |  | 476 |
| K | New York | Northeast | 249 | 248 | 261 | 758 |
| L | New York | Northeast | 248 | 239 | 181 | 668 |
| M | Florida | South | 258 | 194 | 248 | 700 |
| N | Georgia | South | 241 | 250 | 246 | 737 |
| O | North Carolina | South | 231 | 210 |  | 441 |
| P | Tennessee | South |  |  | 248 | 248 |
| Q | Texas | South | 251 | 252 | 266 | 769 |
| R | Texas | South |  |  | 210 | 210 |
| S | Arizona | West |  |  | 249 | 249 |
| T | California | West | 248 | 250 | 249 | 747 |
| U | California | West | 250 | 224 | 199 | 673 |
| V | Colorado | West | 246 | 252 | 251 | 749 |
| W | Utah | West | 248 | 236 | 248 | 732 |
| X | Washington | West | 232 | 247 | 246 | 725 |
| Total |  |  | 4,953 | 4,576 | 4,588 | 14,117 |

**Table S2.** Isolate infection source for all Gram-negative bacilli, Enterobacterales, and *P. aeruginosa* isolates collected by 24 United States laboratories participating in the SMART global surveillance program from 2019 to 2021

|  | *n* (% of total) | | |
| --- | --- | --- | --- |
| Isolate infection source | All Gram-negative bacilli | Enterobacterales | *P. aeruginosa* |
| BSI | 2,937 (20.8) | 2,598 (24.7) | 223 (8.8) |
| IAI | 2,377 (16.8) | 2,088 (19.9) | 233 (9.2) |
| LRTI | 5,706 (40.4) | 3,082 (29.3) | 1,763 (69.8) |
| UTI | 3,036 (21.5) | 2,692 (25.6) | 294 (11.6) |
| None | 61 (0.4) | 49 (0.5) | 11 (0.4) |
| Total | 14,117 (100) | 10,509 (100) | 2,524 (100) |

Abbreviations: BSI, bloodstream infection; IAI, intraabdominal infection; LRTI, lower respiratory tract infection; UTI, urinary tract infection.

**Table S3.** Species distribution of all Gram-negative isolates collected by the SMART global surveillance program in the United States from 2019 to 2021 stratified by isolate-associated parameters

|  |  | | Stratum, *n* (% of total) | | | | | | | |
| --- | --- | --- | --- | --- | --- | --- | --- | --- | --- | --- |
| Species | Overall  (*n*=14,117) | LOS ≥48h  (*n*=4,386) | | LOS <48h  (*n*=7,632) | ICU  (*n*=3,723) | Non-ICU  (*n*=7,522) | BSI  (*n*=2,937) | IAI  (*n*=2,377) | LRTI  (*n*=5,706) | UTI  (*n*=3,036) |
| *Escherichia coli* | 4,154 (29.4) | 917 (20.9) | | 2,642 (34.6) | 711 (19.1) | 2,349 (31.2) | 1,363 (46.4) | 1,003 (42.2) | 570 (10.0) | 1,203 (39.6) |
| *Pseudomonas aeruginosa* | 2,524 (17.9) | 922 (21.0) | | 1,248 (16.4) | 791 (21.2) | 1,325 (17.6) | 223 (7.6) | 233 (9.8) | 1,763 (30.9) | 294 (9.7) |
| *Klebsiella pneumoniae* | 1,930 (13.7) | 603 (13.7) | | 1,025 (13.4) | 510 (13.7) | 1,012 (13.5) | 430 (14.6) | 338 (14.2) | 657 (11.5) | 493 (16.2) |
| *Proteus mirabilis* | 612 (4.3) | 120 (2.7) | | 381 (5.0) | 115 (3.1) | 339 (4.5) | 157 (5.3) | 73 (3.1) | 163 (2.9) | 215 (7.1) |
| *Serratia marcescens* | 563 (4.0) | 222 (5.1) | | 258 (3.4) | 229 (6.2) | 248 (3.3) | 85 (2.9) | 45 (1.9) | 375 (6.6) | 55 (1.8) |
| *Klebsiella oxytoca* | 538 (3.8) | 192 (4.4) | | 267 (3.5) | 166 (4.5) | 284 (3.8) | 105 (3.6) | 114 (4.8) | 230 (4.0) | 86 (2.8) |
| *Stenotrophomonas maltophilia* | 460 (3.3) | 210 (4.8) | | 189 (2.5) | 179 (4.8) | 207 (2.8) | 22 (0.7) | 17 (0.7) | 413 (7.2) | 7 (0.2) |
| *Klebsiella aerogenes* | 445 (3.2) | 205 (4.7) | | 166 (2.2) | 161 (4.3) | 210 (2.8) | 48 (1.6) | 52 (2.2) | 261 (4.6) | 80 (2.6) |
| *Enterobacter cloacae* | 425 (3.0) | 173 (3.9) | | 200 (2.6) | 135 (3.6) | 210 (2.8) | 84 (2.9) | 81 (3.4) | 175 (3.1) | 84 (2.8) |
| *Citrobacter freundii* | 276 (2.0) | 81 (1.8) | | 158 (2.1) | 56 (1.5) | 160 (2.1) | 29 (1.0) | 88 (3.7) | 59 (1.0) | 99 (3.3) |
| Other Enterobacterales species | 1566 (11.1) | 517 (11.8) | | 798 (10.5) | 443 (11.9) | 879 (11.7) | 297 (10.1) | 294 (12.4) | 592 (10.4) | 377 (12.4) |
| Other non-Enterobacterales species | 624 (4.4) | 224 (5.1) | | 300 (3.9) | 227 (6.1) | 299 (4.0) | 94 (3.2) | 39 (1.6) | 448 (7.9) | 43 (1.4) |

Abbreviations: LOS, length of hospital stay at time of specimen collection; BSI, bloodstream infection; IAI, intraabdominal infection; LRTI, lower respiratory tract infection; UTI, urinary tract infection.

**Table S4.** Cross-susceptibility to ceftolozane/tazobactam, imipenem/relebactam, and ceftazidime/avibactam among NME isolates with ceftolozane/tazobactam-nonsusceptible, imipenem/relebactam-nonsusceptible, and ceftazidime/avibactam-resistant phenotypes

|  |  | Antimicrobial Agent, % Susceptible | | |
| --- | --- | --- | --- | --- |
| Phenotype | *n* (% of all isolates) | C/T | IMR | CZA |
| C/T-NS | 587 (6.2) | 0 | 96.1 | 97.6 |
| IMR-NS | 106 (1.1) | 78.3 | 0 | 92.5 |
| CZA-R | 14 (0.1) | 0 | 42.9 | 0 |

Abbreviations: C/T, ceftolozane/tazobactam; IMR, imipenem/relebactam; CZA, ceftazidime/avibactam; NS, non-susceptible; R, resistant.

**Table S5.** Antimicrobial susceptibility testing results for isolates of Enterobacterales, NME, and *P. aeruginosa* collected by the SMART global surveillance program in the United States from 2019 to 2021 stratified by census region

|  |  | % Susceptible | | | | | | | | | | |
| --- | --- | --- | --- | --- | --- | --- | --- | --- | --- | --- | --- | --- |
| Organism  Census region | *n* | C/T | IMI/REL | CZA | IMI | MEM | P/T | FEP | CAZ | ATM | LVX^a^ | AMK^b^ |
| Enterobacterales |  |  |  |  |  |  |  |  |  |  |  |  |
| Midwest | 3,860 | 95.1 | NA | 99.9 | 88.5 | 99.0 | 89.4 | 90.6 | 86.9 | 87.2 | 80.1 | NA |
| Northeast | 1,391 | 94.2 | NA | 99.9 | 86.3 | 99.1 | 88.2 | 90.7 | 87.3 | 87.1 | 81.3 | NA |
| South | 2,360 | 95.7 | NA | 99.9 | 88.5 | 99.3 | 88.6 | 87.8 | 84.5 | 83.8 | 79.8 | NA |
| West | 2,898 | 91.9 | NA | 99.6 | 89.0 | 98.8 | 84.9 | 87.0 | 82.6 | 82.0 | 78.9 | NA |
| NME |  |  |  |  |  |  |  |  |  |  |  |  |
| Midwest | 3,448 | 94.6 | 99.1 | 99.9 | 95.8 | 99.0 | 88.5 | 90.0 | 86.2 | 86.0 | 82.0 | NA |
| Northeast | 1,243 | 93.7 | 98.5 | 99.8 | 93.7 | 99.0 | 86.9 | 90.1 | 86.5 | 85.8 | 82.1 | NA |
| South | 2,167 | 95.4 | 98.8 | 99.9 | 94.6 | 99.2 | 87.8 | 87.0 | 83.5 | 82.6 | 80.6 | NA |
| West | 2,666 | 91.6 | 98.9 | 99.7 | 94.5 | 98.6 | 84.1 | 86.5 | 82.1 | 80.8 | 80.2 | NA |
| *P. aeruginosa* |  |  |  |  |  |  |  |  |  |  |  |  |
| Midwest | 949 | 96.8 | 92.8 | 96.5 | 68.3 | 80.7 | 81.9 | 84.3 | 82.6 | 74.1 | 65.5 | 95.6 |
| Northeast | 361 | 96.7 | 87.5 | 93.6 | 58.4 | 71.7 | 72.0 | 77.3 | 74.5 | 65.1 | 64.6 | 97.8 |
| South | 558 | 97.3 | 91.2 | 95.7 | 64.0 | 83.0 | 78.0 | 83.2 | 82.3 | 71.1 | 71.7 | 98.2 |
| West | 656 | 95.7 | 88.9 | 92.2 | 65.1 | 78.7 | 77.9 | 81.6 | 80.0 | 70.0 | 66.3 | 97.7 |

Abbreviations: C/T, ceftolozane/tazobactam; IMI/REL, imipenem/relebactam; CZA, ceftazidime/avibactam; IMI, imipenem; MEM, meropenem; P/T, piperacillin/tazobactam; FEP, cefepime; CAZ, ceftazidime; ATM, aztreonam; LVX, levofloxacin; AMK, amikacin; NME, non-*Morganellaceae* Enterobacterales; NA, not available.

^a^ Susceptibility to levofloxacin was not available for *Salmonella* spp. (*n*=17) because the tested concentration range did not extend low enough for the *Salmonella*-specific CLSI susceptible breakpoint for that agent.

^b^ Susceptibility to amikacin was not determinable for Enterobacterales because the tested concentration range did not extend low enough to include the revised 2023 CLSI susceptible breakpoint for that agent.

**Table S6.** Cross-susceptibility to ceftolozane/tazobactam, imipenem/relebactam, and ceftazidime/avibactam among *P. aeruginosa* isolates with ceftolozane/tazobactam-nonsusceptible, imipenem/relebactam-nonsusceptible, and ceftazidime/avibactam-resistant phenotypes

|  |  | Antimicrobial Agent, % Susceptible | | |
| --- | --- | --- | --- | --- |
| Phenotype | *n* (% of all isolates) | C/T | IMR | CZA |
| C/T-NS | 85 (3.4) | 0 | 51.8 | 42.4 |
| IMR-NS | 235 (9.3) | 82.6 | 0 | 68.9 |
| CZA-R | 131 (5.2) | 62.6 | 44.3 | 0 |

Abbreviations: C/T, ceftolozane/tazobactam; IMR, imipenem/relebactam; CZA, ceftazidime/avibactam; NS, non-susceptible; R, resistant.
